# Silencing Heat Shock Protein 27 Inhibits the Progression and Metastasis of Colorectal Cancer (CRC) by Maintaining the Stability of Stromal Interaction Molecule 1 (STIM1) Proteins

**DOI:** 10.3390/cells7120262

**Published:** 2018-12-10

**Authors:** Chien-Yu Huang, Po-Li Wei, Wei-Yu Chen, Wei-Chiao Chang, Yu-Jia Chang

**Affiliations:** 1 Department of Surgery, College of Medicine, Taipei Medical University, Taipei 110, Taiwan; cyh@tmu.edu.tw (C.-Y.H.); poliwei@tmu.edu.tw (P.-L.W.); 2 Division of General Surgery, Department of Surgery, Shuang Ho Hospital, Taipei Medical University, Taipei 110, Taiwan; 3 Division of Colorectal Surgery, Department of Surgery, Wan Fang Hospital, Taipei Medical University, Taipei 110, Taiwan; 4 Cancer Research Center and Translational Laboratory, Department of Medical Research, Taipei Medical University Hospital, Taipei Medical University, Taipei 110, Taiwan; 5 Division of Colorectal Surgery, Department of Surgery, Taipei Medical University Hospital, Taipei Medical University, Taipei 110, Taiwan; 6 Graduate Institute of Cancer Biology and Drug Discovery, Taipei Medical University, Taipei 110, Taiwan; 7 Department of Pathology, School of Medicine, College of Medicine, Taipei Medical University, Taipei 110, Taiwan; wychen.patho@gmail.com; 8 Department of Pathology, Wan Fang Hospital, Taipei Medical University, Taipei 110, Taiwan; 9 School of Pharmacy, Taipei Medical University, Taipei 110, Taiwan; weichiao.chang@gmail.com; 10 Master Program for Clinical Pharmacogenomics and Pharmacoproteomics, Taipei Medical University, Taipei 110, Taiwan; 11 Graduate Institute of Clinical Medicine, College of Medicine, Taipei Medical University, Taipei 110, Taiwan

**Keywords:** HSP27, ER, SOCE, STIM1, CRC

## Abstract

The incidence of colorectal cancer (CRC) has significantly increased in recent decades, and this disease has become an important health issue worldwide. Currently, there is no useful prognostic or diagnostic biomarker for CRC. Heat shock protein 27 (HSP27) is a chaperone that interacts with many proteins. HSP27 has been shown to be overexpressed in many cancers, including colon cancer, and its overexpression is related to poor disease outcome. Although the importance of HSP27 as a biomarker cannot be underrated, its detailed mechanisms in colon cancer are still unclear. In vitro studies have indicated that silencing HSP27 reduces the proliferation, migration and invasion of colon cancer cells, and xenograft models have shown that silencing HSP27 decreases tumor progression. Tissue array results showed that colon cancer patients with high expression of HSP27 exhibited poor prognosis. In addition, we found a reduction of calcium influx through a decrease in STIM1 protein after HSP27 was abolished. The formation of puncta was decreased in HSP27 knockdown (HSP27KD) cells after thapsigargin (TG) treatment. Finally, we confirmed that the reduction of STIM1 after HSP27 silencing may be due to a loss of STIM1 stability instead of transcription. HSP27 may interact with STIM1 but not Orai1, as shown by immunoprecipitation assays. HSP27 and STIM1 were co-expressed in CRC specimens. Our study showed that HSP27 is a key mediator in the progression and metastasis of CRC by regulating the store-operated calcium entry. This novel pathway may provide a new direction for development of therapeutic strategies for CRC.

## 1. Introduction

Colorectal cancer (CRC) is the second most common type of life-threatening cancer worldwide [1]. CRC is the third and the second most common malignant cancer in men and women, respectively [1,2]. Surgery followed by chemotherapy is a major method for treatment of CRC, but this is limited by the accompanying toxic side effects [3,4,5]. The identification of less toxic anticancer agents will improve the management of CRC and may reduce treatment costs by providing better treatment options. In the past decade, a significant increase in the knowledge of CRC’s molecular genetics has led to the identification of several molecules as prognostic, predictive and therapeutic biomarkers [6,7,8].

Heat shock protein 27 (HSP27) belongs to the small HSP (15–30 kDa) family of mammalian heat shock proteins, which are classified into four main families based on molecular weights [9,10]. Molecular chaperones are responsible for maintaining the correct conformation of other proteins during protein-protein interactions [11]. During stress conditions, such as malignancy, HSP27 is upregulated as a key driver of the adaptive stress-response pathway, a mechanism of adaptation to different stress conditions in cancer cells [12]. This protein is highly expressed in a variety of human cancers, such as breast, ovarian, gastric, prostate, endometrial, liver, bladder, and colon cancers and leukemia [13,14,15,16]. The upregulation of HSP27 is associated with aggressive and malignant properties of cancer cells, apoptosis inhibition, resistance to chemotherapy or radiotherapy, and hence, a poor prognosis [17,18,19,20]. Inhibitors targeting HSP27, such as chlorpromazine, acepromazine, or apatorsen (OGX-427) showed acceptable adverse event in phase-Ι human study [21,22]. However, the mechanisms for HSP27 in CRC progression are still unclear.

Calcium channels are important for the regulation of cellular metabolism [23]. The critical role of Ca^2+^ and Ca^2+^ ion channels in accelerating proliferation, invasion and metastasis in various cancers has been emphasized in multiple studies [24,25,26]. Ca^2+^ signaling coordination is controlled by the endoplasmic reticulum (ER) Ca^2+^ sensor, stromal interaction molecule 1 (STIM1), which regulates the Ca^2+^ entry process in response to external stimuli [27]. Previous studies have suggested a critical role of STIM1 in tumorigenesis [28,29]. Targeting of STIM1 by knockdown or pharmacological inhibition of the store-operated calcium entry (SOCE) was shown to suppress tumor metastasis in melanoma, hepatocellular carcinoma, colorectal cancer and breast cancer [30,31,32,33] and sensitized cancer cells’ response to therapy [34]. However, no report has examined the role of HSP27 in regulation of Ca^2+^ ion channels.

In the current study, we demonstrated that high HSP27 levels were correlated with a poor outcome in CRC patients. Targeting HSP27 suppressed cancer progression and metastasis. In addition, HSP27 was co-expressed with STIM1 and interacted with STIM1 to maintain the stability of STIM1 proteins, indicating that targeting HSP27 may be a new direction for CRC therapy. These findings provide new insight into the possibility of targeting HSP27 for CRC therapy.

## 2. Materials and Methods

### 2.1. Chemicals, Reagents, and Cell Culture

Propidium iodide (PI), Tris–HCl, trypan blue, ethylenediaminetetraacetic acid (EDTA), sulforhodamine B (SRB), ribonuclease A, thapsigargin (TG), cell lysis buffer, and dimethyl sulfoxide (DMSO) were obtained from Sigma Chemical (St. Louis, MO, USA). Anti-GAPDH (SC-32233), Fibronectin (SC-9068), vimentin (SC-6260), Orai1 (SC-68895), p21 (SC-397), p27 (SC-528), cyclin D (SC-2004), and cyclin E (SC-481) antibodies were purchased from Santa Cruz Biotechnology (Santa Cruz, CA, USA). Anti-STIM1 antibody was from BD Biosciences (San Jose, CA, USA). Anti *N*-cadherin (13116s) and anti-E-cadherin (3195s) antibodies were purchased from Cell Signaling (Danvers, MA, USA). Anti-cyclin B and cyclin A2 antibody was purchased from Genetex (Irvine, CA, USA). Anti-HSP27 antibody was purchased from Abcam (Cambridge, UK). HCT116, HT-29 and DLD-1 cell lines were purchased from the American Type Culture Collection (ATCC, Rockville, MD, USA). The cells were cultured in RPMI with 10% fetal bovine serum (FBS) in a humidified incubator (37 °C, 5% CO_2_). The cells were either sub-cultured or used before they reached 80% confluence.

### 2.2. Tissue Samples and Immunohistochemistry

Two formalin-fixed paraffin-embedded tissue microarray sections including a total of 177 cases of primary colon adenocarcinoma (catalog no. HCol-Ade180Sur-02 and HCol-Ade180Sur-03) were obtained from US Biomax, Inc. (Rockville, MD, USA). Each case contained one core of adenocarcinoma tissue and one core of non-neoplastic colon tissue. The immunohistochemical staining was performed as previously described [33]. Briefly, the deparaffinized sections were treated with 3% hydrogen peroxide, followed by heat-induced antigen retrieval in citric acid buffer (pH 6.0) at 121 °C for 10 min in a decloaking chamber (Biocare Medical, Concord, CA, USA). The sections were incubated with HSP27 antibody (catalog no. ab2790, 1:750; Abcam plc, Cambridge, UK) at 4 °C overnight. HSP27 expression was then examined by the Starr Trek Universal HRP Detection System (Biocare Medical., Concord, CA, USA). The intensity of HSP27 expression was scored semiquantitatively as weakly positive, moderately positive or strongly positive as previously described [6]. For determination of the association of HSP27 and STIM1 expression levels, two consecutive formalin-fixed paraffin-embedded tissue microarray sections purchased from SuperBioChips Laboratories (catalog no. CDA3, Seoul, Korea) were stained with HSP27 and STIM1 (catalog no. HPA012123, 1:200, Atlas Antibodies AB, AlbaNova University Center, Stockholm, Sweden) by immunohistochemistry using the aforementioned protocol. In total, 40 cases of primary colon adenocarcinoma were included in this tissue microarray section. The evaluation of HSP27 and STIM1 expression levels was performed using the H-score as described previously [34]. Briefly, all tumor cells in each tissue core were analyzed. The intensity of the staining was denoted as 0 (negative), 1+ (weakly positive), 2+ (moderately positive) or 3+ (strongly positive). The percentage of the tumor cells at each intensity was determined. The H-score value (range of 0–300) was calculated by summation of the intensity of the staining (0 to 3) multiplied by the corresponding percentages of the cells at each intensity (0–100). 

### 2.3. HSP27 Expression Manipulation in Colon Cancer Cells

The expression of HSP27 was ablated in CRC cells using short hairpin RNA (shRNA) purchased from the National RNAi Core Facility, Academia Sinica, Taiwan, as previously described [6]. Briefly, the plasmids containing human HSP27-specific shRNA and control shRNA were transfected into CRC cells and selected by puromycin to generate stably transfected cells, as described previously [35,36]. For overexpression of HSP27, the pCMV6-XL5-HSP27 and pCMV6-XL5 plasmids were purchased from OriGene Technologies, Inc. (Rockville, MD, USA). The plasmids were transfected into cells. HSP27 expression was verified by quantitative real-time PCR and western blot analyses. 

### 2.4. Protein Extraction and Western Blot Analysis

Target proteins were revealed by sodium dodecyl sulfate-polyacrylamide gel electrophoresis (SDS-PAGE) and western blot analysis. The cells were lysed using cell lysis buffer (Sigma-C2978) with protease inhibitors (Complete Protease Inhibitor Tablets; Boehringer Mannheim, Indianapolis, IN, USA). The proteins were separated using 10% SDS-PAGE and electrotransferred onto polyvinylidene difluoride (PVDF) membranes. The membranes were incubated overnight at 4 °C with a primary antibody against a specific target and chemiluminescent detection was uses a horseradish peroxidase-conjugated secondary antibody (1:5000). The signals were visualized with a chemiluminescence reagent (GE Healthcare Life Sciences, Pittsburgh, PA, USA) and detected using the VersaDoc 5000 system (Bio-Rad Laboratories, Hercules, CA, USA) [33].

### 2.5. Cell Cycle Analysis by Flow Cytometry

Cells (3 × 10^5^) were seeded onto 6-well plates overnight and then subjected to starvation for 18 h. The medium was replaced by 10% FBS/RPMI. After 18 h of recovery, the cells were harvested, washed with PBS, and fixed in 75% ethanol overnight at 4 °C. Then the cells were treated with RNase A (at a final concentration of 40 μg/mL), and the cellular DNA content was analyzed using a flow cytometer (BD Biosciences, San Jose, CA, USA), following staining with PI (40 μg/mL) for 30 min at room temperature. The DNA content was quantified using Modfit software (Verity Software House, Topsham, ME, USA). 

### 2.6. Evaluation of Cell Proliferation and Cell Migration Using the Xcelligence Biosensor System

The cell proliferation and migration were assessed by real-time monitor system with 16-well plates (E-plate 16 for proliferation and CIM-plate 16 for migration, ACEA BioSciences, Inc., San Diego, CA, USA) and the curves were monitored by an RTCA DP instrument (ACEA BioSciences, Inc., San Diego, CA, USA). For proliferation, cells were seededat 10,000 cells/well in FBS-containing medium. The plate was then monitored once every 30 s for 4 h and once every half hour thereafter. For evaluation of migration, 10% FBS RPMI medium was added to the lower chamber, then cells were seeded into the upper chamber at 20,000 cells/well with serum-free medium. The cell migratory activity was monitored every 10 s for 40 min and once every hour. The data were analyzed using RTCA software 1.2 (ACEA BioSciences, Inc., San Diego, CA, USA) [37,38].

### 2.7. SRB Colorimetric Assay for Cytotoxicity Screening Stain

Cells (2 × 10^4^) were seeded into 24-well plates and after the specific time intervals, cells were fixed with 10% trichloroacetic acid overnight at 4 °C and then stained with the 0.4% *w*/*v* protein-bound SRB for 30 min at room temperature. Next, the stained cells were washed twice with 1% acetic acid. After air-drying, the protein-bound dye was dissolved in 10 mM Tris base solution for OD measurement at 515 nm by using a microplate reader (Bio-Rad Laboratories, Hercules, CA, USA).

### 2.8. In Vivo Tumor Xenograft Experiments

All mouse experiments were conducted in strict accordance with the regulations of the Institutional Animal Care and Use Committee (IACUC), Taipei Medical University (LAC-2015-0246). Male nude mice (5 weeks old) were used as the in vivo experimental model. The scrambled control and HSP27KD DLD-1 cells were suspended in PBS and a volume of 0.1 mL with 1 × 10^6^ cells was injected subcutaneously (s.c.) into the left side of each mouse. Tumor dimensions and body weights were recorded twice per week. Tumor volume based on caliper measurements were calculated using the formula (L × w^2^)/2, where L and w are the larger and smaller tumor dimensions, respectively [39]. After 5 weeks, the mice were sacrificed, and all the tumors were excised and weighed. The excised tumor tissue was fixed in 10% formalin and embedded in paraffin for immunohistochemical staining or snap-frozen in liquid nitrogen for further evaluation. 

### 2.9. Transwell Migration Assay and Invasion Assay

In vitro cell migration and invasion were examined using a BD Falcon cell culture insert and a BD BioCoat™ Matrigel Invasion Chamber (BD Biosciences, San Jose, CA, USA), as described in previous study [40,41]. Aliquots of 1 × 10^5^ cells were in 500 μL of serum-free RPMI and were seeded into the upper compartments of each chamber. The lower compartments were added 1 mL of RPMI with 10% FBS. After incubation for 48 h at 37 °C in 5% CO_2_, the non-migrating and non-invading cells were removed from the upper surface of the membrane by scrubbing. The cells on the reverse side were stained with 0.1% crystal violet, and then counted under a microscope at 100× magnification.

### 2.10. Determination of Calcium Concentrations

The DLD-1 cells (scrambled control and HSP27KD) were trypsinized and seeded on glass coverslips within 6-well culture plates. After 36 h of incubation, cells were washed and stained with 1 μM of Fluo-4 AM (Sigma-Aldrich, St. Louis, MO, USA) for up to 30 min. Following the staining step, coverslips with dye-loaded cells were washed and mounted in a microscope chamber, which was then filled with calcium-free buffer. During the measurement, TG (Sigma-Aldrich, St. Louis, MO, USA) and 2 mM of calcium buffer were added at 60 and 330 s to trigger calcium release from the ER and extracellular calcium influx, respectively. Real-time intracellular calcium signals were determined based on the fluorescence intensities of Fluo-4 AM by an inverted fluorescence microscope (Leica, Wetzlar, Germany) equipped with sCMOS camera (Andor Technology, Belfast, UK) and MetaFluor software (Molecular Devices, San Jose, CA, USA).

### 2.11. Immunocytochemistry

The DLD-1 cells were fixed in 4% paraformaldehyde for 15 min at RT and incubated with blocking buffer (5% BSA). The cells were then incubated with primary antibody and subsequently incubated with Alexa Fluor 488/594 goat anti-rabbit/mouse IgG (Sigma-Aldrich, St. Louis, MO, USA). After immunostaining, the images were taken using a Leica TCS SP5 Confocal Spectral Microscope Imaging System (Leica Microsystems, Wetzlar, Germany) or an Olympus microscope and DP80 microscope camera (Olympus Corporation, Tokyo, Japan).

### 2.12. Immunoprecipitation Assay

The immunoprecipitation assays were performed according to the Catch and Release v2.0 Kit (Millipore Merck, Burlington, MA, USA) recommendations. Briefly, cell lysates containing 500 μg protein were incubated with primary antibodies for HSP27 (Cell Signaling Tech, Danvers, MA, USA) at 4 °C overnight on a rotator by using Catch and Release spin columns. Then, the columns were washed 3 times with wash buffer and centrifuged at 2000 g, 15–30 s for each wash. Protein bound to the beads was eluted by elution buffer and analyzed by western blot analyses.

### 2.13. RT-PCR and Quantitative RT-PCR Analysis

Total RNA was extracted using TRIzol reagent according to the manufacturer’s instructions (Invitrogen Life Technologies, Carlsbad, CA, USA). Then total RNA (8 μg) was used for RT reactions in a 20 μL reaction to synthesize cDNA using a cDNA Synthesis Kit (Invitrogen Life Technologies). The gene expression was monitored by quantitative RT-PCR performed using the Power SYBR-Green real-time RT-PCR system and an ABI 7500 FASTTM detection system (Applied Biosystems, Foster City, CA, USA). The quantitative PCR conditions were as follows: 95 °C for 10 min followed by 40 cycles of 95 °C for 15 s and 60 °C for 1 min. A melting curve was conducted after the PCR cycles, followed by a cooling step. Each sample was assayed in triplicate in each experiment, and each experiment was repeated 3 times. Quantitative target gene expression was normalized to the expression levels of *GAPDH* gene. All primer sequences are listed in Table 1.

### 2.14. Statistical Analysis

In the tissue array results, the associations between the clinicopathologic characteristics and HSP27 expression in colon adenocarcinoma were determined using a Chi-square test. An overall survival curve was calculated using the Kaplan-Meier method and evaluated by log-rank tests. The correlation between HSP27 and STIM1 expression levels was evaluated using Pearson’s correlation coefficient. *p* < 0.05 was considered statistically significant. All statistical calculations were performed using SPSS 18.0 statistical software (SPSS Inc., Chicago, IL, USA). The experiments were repeated at least three times independently. All data collected from QPCR and cell proliferation, migration, invasion experiments are expressed as the mean ± SD. The data presented in some figures are derived from a representative experiment that was quantitatively similar to the replicate experiments. Statistical significance was examined using a two-tailed Student’s *t* test. Asterisks in the figures indicate significant differences between the indicated experimental groups and the corresponding control conditions (*p* < 0.05, see figure legends).

## 3. Results

### 3.1. The HSP27 Expression and Clinicopathological Significance in Colon Adenocarcinoma

**Figure 1:**
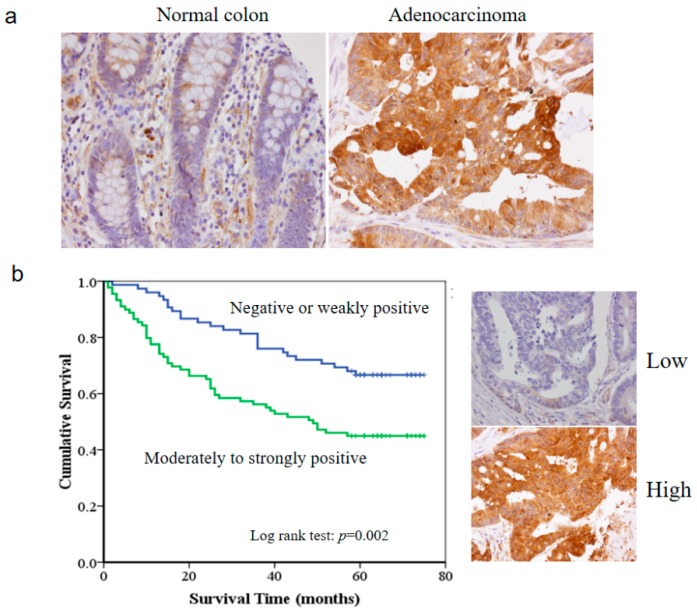
The HSP27 expression level correlates with clinicopathologic characters in colon adenocarcinoma. (**a**) HSP27 expression was higher in colon adenocarcinoma tissue than non-neoplastic colon tissue (*p* = 0.001). (**b**) Moderate/strong HSP27 expression was linked with poorer prognosis than weak HSP 27 expression (*p* = 0.002).

To elucidate the biological significance of HSP27 in colon adenocarcinoma, we performed immunohistochemical staining for HSP27 on two tissue microarray sections that included 177 cases of colon adenocarcinoma. The associations between HSP27 expression and clinicopathological characteristics of CRC are shown in Table 2. Carcinoma tissues tended to express higher levels of HSP27 than the non-neoplastic colon tissues (*p* = 0.001) (Figure 1a). Invasive severity of the adenocarcinoma (T status of TNM staging) was also associated with HSP27 expression (*p* = 0.009). Deeper invasion was more commonly observed in the adenocarcinomas with high HSP27 expression than those with low expression. Level of HSP27 expression showed no correlation with gender, patient age, lymph node metastasis, and distant metastasis. Kaplan-Meier curve analysis demonstrated that the overall survival of the CRC patients with high HSP27 expression was remarkable worse than that of the patients with low HSP27 expression (*p* = 0.002) (Figure 1b). 

### 3.2. HSP27 Expression and Roles in Cell Growth of CRC Cell Lines

**Figure 2:**
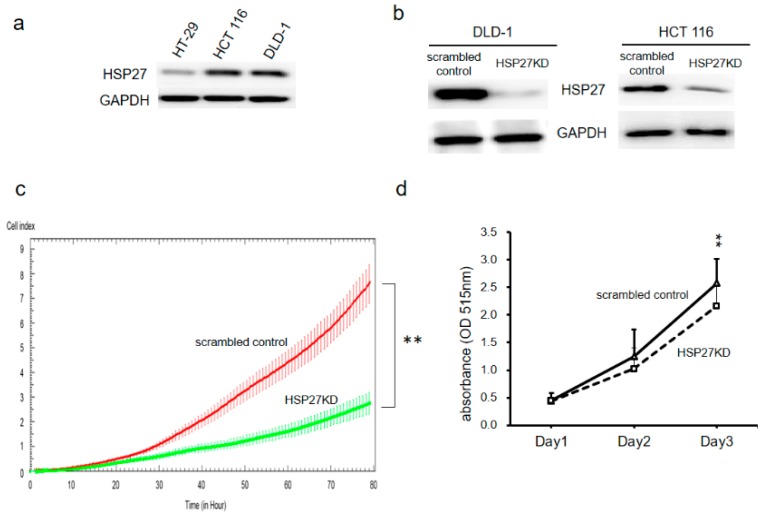
Targeting HSP27 reduced the cell proliferation activity. (**a**) HSP27 expression levels in CRC cells were determined by western blot analyses. HSP27 knockdown (HSP27KD) cells were generated by HSP27-specific shRNA, and stably transfected cells were selected by antibiotics. (**b**) The levels of HSP27 in the scrambled control and HSP27KD cells were determined. (**c**) The proliferation of scrambled control and HSP27KD DLD-1 cells was determined by the xCELLigence biosensor system. (**d**) The cell survival rates of scrambled control and HSP27KD HCT116 cells were determined by the SRB method. All experiments were independently repeated three times. ** indicates *p* < 0.005.

To clarify the role of HSP27 in CRC, we exanimated the expression levels of HSP27 in four different human CRC cell lines (HT-29, HCT116 and DLD-1). HT-29 expressed lower levels of HSP27 than the more malignant DLD-1 cell line, which expressed high levels of HSP27 (Figure 2a). The expression levels of HSP27 were then knocked down in DLD-1 and HCT116 cells by the shRNA method. As shown in Figure 2b, HSP27 levels were reduced over 85% in HSP27-specific shRNA-transfected (HSP27KD) DLD-1 and HCT116 cell lines compared with control shRNA-transfected cells (scrambled control). 

### 3.3. Silencing HSP27 Decreased Cell Proliferation via Cell Cycle Arrest

The growth of control shRNA-transfected (scrambled control) and HSP27KD cells was measured by a biosensor system or SRB assays. As shown in Figure 2c,d, knockdown of HSP27 in both cell lines was associated with decreased cell proliferation compared with that of the scrambled controls. These results indicate that HSP27 plays a role in CRC growth activity.

### 3.4. Silencing HSP27 Caused Cell Cycle Arrest at S phase

**Figure 3:**
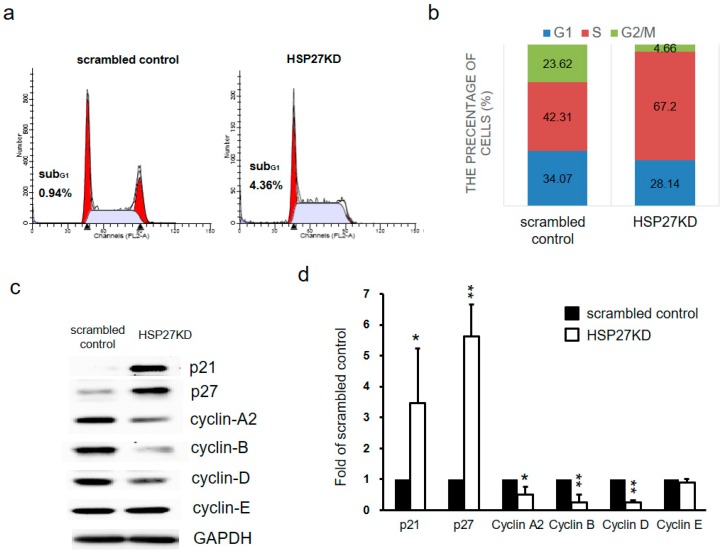
Targeting HSP27 influenced cell cycle distribution. (**a**) The cell cycle distribution between scrambled control and HSP27KD DLD-1 cells was determined by PI staining followed by flow cytometry analysis. (**b**) The cell cycle distribution was plotted. An increase in the S cell population was found in HSP27KD cells. (**c**) Cell cycle-regulated proteins (p21, p27, cyclin-A2, B, D, and E) were detected by western blot analyses. GADPH was the internal control. (**d**) The signal of target proteins was quantified and normalized to that of the internal control. The number of scrambled control samples was adjusted to 1. All experiments were independently repeated three times. * indicates *p* < 0.05, ** indicates *p* < 0.01.

To elucidate the decrease in cell growth activity, we performed an analysis of cell cycle distribution between the scrambled control and HSP27KD by flow cytometry after PI staining. As shown in Figure 3a,b, the population of subG1 cells did not change in HSP27KD cells. Interestingly, the population of HSP27KD cells at G2-M phase was decreased and increased significantly at S phase. To further determine the effect of S phase arrest on HSP27KD cells, we measured the expression levels of cell cycle regulatory proteins by western blot analyses. As shown in Figure 3c,d, the levels of cyclin-A2, B and D were decreased in HSP27KD cells compared to the scrambled control cells, but cyclin E did not show significant changes. The HSP27KD cells also showed upregulation of p21 and p27 (Figure 3c,d). These results indicate that silenced HSP27 causes S phase arrest through changes in S phase checkpoint proteins, cyclins (A2, B, D), p21 and p27.

### 3.5. HSP27 Regulated Cancer Progression in Xenograft Mouse Models

**Figure 4:**
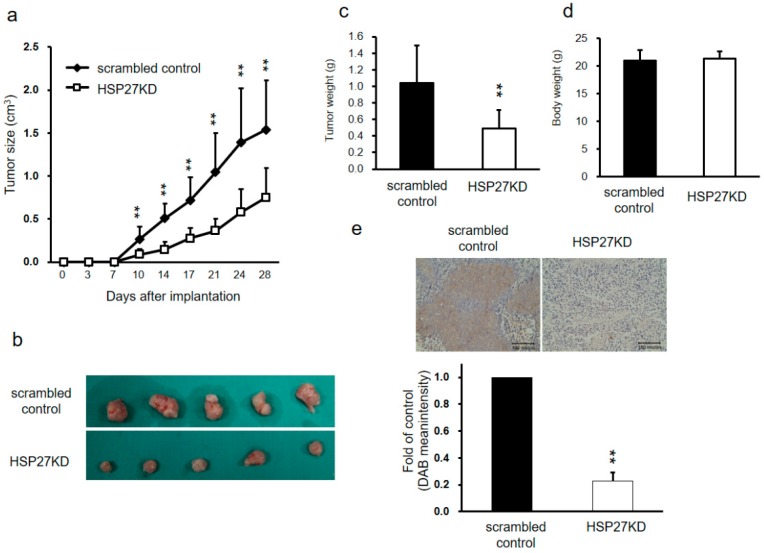
Silenced HSP27 reduced the tumor formation in a xenograft model. (**a**) Scrambled control and HSP27KD DLD-1 cells were implanted subcutaneously into 5-week-old male nude mice. The tumor size was measured twice per week. The plot indicates the tumor volume vs time. Mice were sacrificed on day 28. (**b**) The scrambled control group had larger tumors than the HSP27KD group. (**c**) Tumor weights were plotted and showed that HSP27KD has lower tumor weights than those of the control group. (**d**) The body weight was measured twice per week. At day 28, the body weights of scrambled control and HSP27KD mice were measured and plotted. Similar body weights between the scrambled control and HSP27KD groups. (**e**) HSP27 expression was lower in HSP27KD tumors than in control tumors. There were 5 mice in each group. ** indicates *p* < 0.005.

To further validation the in vitro results, we used a xenograft mouse model to clarify the role of HSP27 in CRC tumorgenesis. 1 × 10^6^ DLD-1 cells of scrambled control or HSP27KD group were injected into the left side flank of nude mice. The body weight and tumor volume of the mice were evaluated twice per week. The tumor sizes and growth rates in the HSP27KD group were significantly decreased compared to those in the scrambled control group (Figure 4a,b). The tumor weights in the HSP27KD group were dramatically reduced (Figure 4c). However, there was no significant difference in body weights between the HSP27KD mice and the scrambled control mice (Figure 4d). The tumors were then harvested, and immunohistochemical staining was performed to evaluate HSP27 expression levels. We found that HSP27 expression was lower in HSP27KD mouse tumors than in control tumors (Figure 4e). Our results indicate that HSP27 influences CRC tumorgenesis in vivo xenograft model.

### 3.6. Silencing HSP27 Expression Inhibited Cell Migration and Invasion of CRC cells

**Figure 5:**
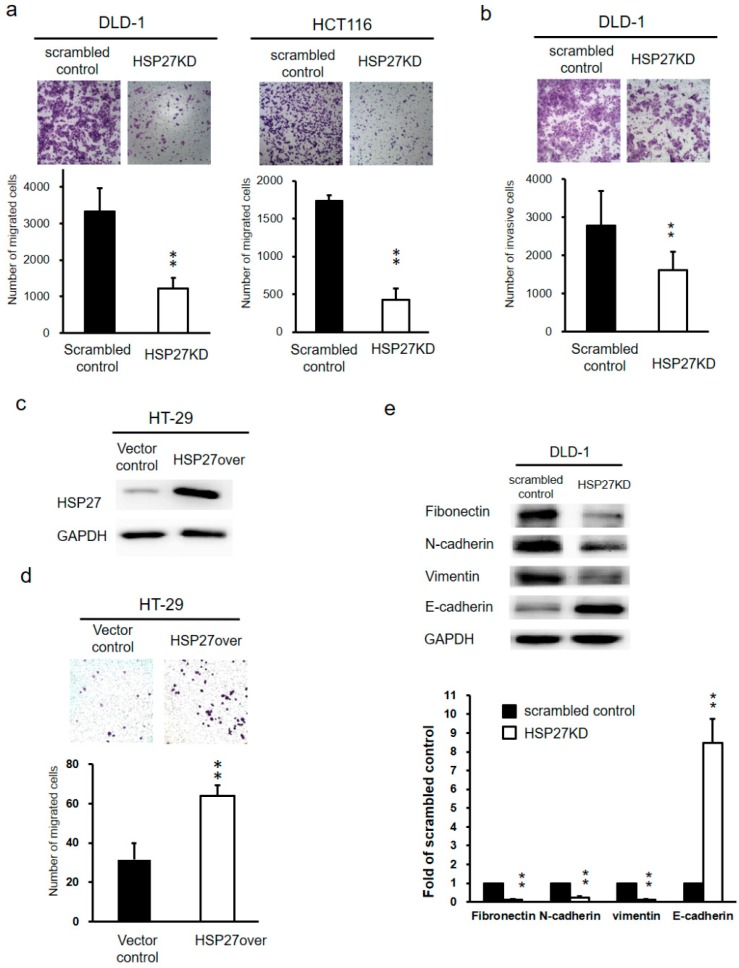
Silenced HSP27 suppressed the migratory and invasive abilities of CRC cells. The migratory (**a**) or invasive (**b**) ability was determined in scrambled control and HSP27KD CRC cells. (**c**) HSP27 was overexpressed in HT-29 cells. HSP27 levels were determined by western blot analyses. (**d**) Overexpression of HSP27 in HT-29 cells enhanced the migratory ability. (**e**) EMT-related biomarkers (Fibronectin, *N*-cadherin, vimentin, E-cadherin) were determined by western blot analyses in the scrambled control and HSP27KD cells. The signal of target proteins was quantified and normalized to that of the internal control. The number of scrambled control samples was adjusted to 1. All experiments were independently repeated three times. ** indicates *p* < 0.01.

The ability of migration in CRC cells after HSP27 knockdown was evaluated with transwell migration assay. The migratory ability was decreased in HSP27KD DLD-1 and HCT116 cells compared to scrambled control cells (Figure 5a). The quantitative results showed that number of migrated cells was decreased by over 50% in HSP27KD groups compared to the scrambled control groups, both DLD-1 and HCT116 cells. To evaluate the invasive ability of scrambled control and HSP27KD cells, we performed invasion assays. The number of invasive cells in HSP27KD cells was remarkable reduced compared to that in scrambled control cells (*p* < 0.01) for DLD-1 cells (Figure 5b), indicating that HSP27 plays an important role in the invasiveness of CRC cells. On the other hand, we overexpressed HSP27 in HT-29 cells and observed higher HSP27 levels in HSP27-overexpressing (HSP27over) HT-29 cells than vector control cells (Figure 5c). Migration assays showed that the number of migratory cells was increased dramatically in HSP27over HT-29 cells (Figure 5d). These results indicate that HSP27 level may influence the metastasis of CRC cells.

### 3.7. Silencing HSP27 Influenced the Epithelial-Mesenchymal Transition (EMT)

The EMT is a crucial process in the aggressiveness of different types of cancers. Thus, to dissect the mechanism by which HSP27 knockdown suppresses metastasis of CRC, we examined the expression levels of EMT markers (Fibronectin, *N*-cadherin, vimentin, and E-cadherin). As shown in Figure 5e, the expression levels of mesenchymal markers (Fibronectin, *N*-cadherin, and vimentin) were reduced, while the expression of the epithelial marker E-cadherin was increased after silencing HSP27. These results indicated that HSP27 may mediate cancer aggressiveness through the EMT transformation.

### 3.8. Silencing HSP27 Expression Abolished Calcium Channel Signals

**Figure 6:**
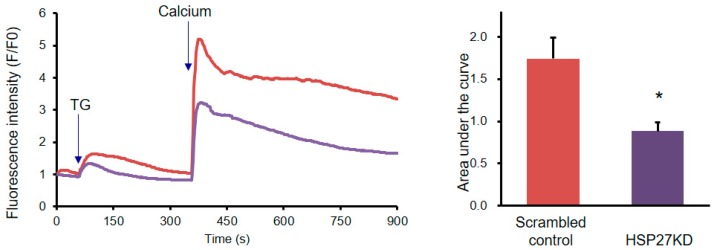
HSP27 mediated TG-induced intracellular calcium mobilization. (**a**) Representative intracellular Ca^2+^ ([Ca^2+^]i) measurement in scrambled control and HSP27KD cells. Each trace is the mean [Ca^2+^]i measurement of at least 100 cells. The SOCE amplitude indicates the rise of [Ca^2+^]i in the replenishment of [Ca^2+^]o from 0 to 2 mM. Arrow, adding 2 μM thapsigargin (TG). (**b**) Quantitative analysis of SOCE. Each value represents the mean ± SD of at least 100 cells. * *p* < 0.01, by *t* tests. Data are the mean ± SD of three independent experiments.

Calcium signals are believed to regulate various functions of the cell, such as motility, secretion, proliferation, gene expression, cell survival, and programmed cell death [42]. Under experimental settings, a microsomal calcium ATPase inhibitor, TG, can be used to increase calcium [42,43]. However, there is little information about the role of HSP27 in calcium channel signaling. Therefore, scrambled control and HSP27KD cells were stimulated with 2 μM TG in a calcium-free solution, and induced short-term calcium release. After an add-back protocol of addition of 2 mM calcium solution, calcium influx signals were detected. Application of 2 μM TG to scrambled control and HSP27KD cells stimulated *HSP27* gene expression in a time-dependent manner. As shown in Figure 6, the stimulation of calcium influx was reduced in HSP27KD cells. 

### 3.9. Silencing HSP27 Reduced STIM1 Levels

**Figure 7:**
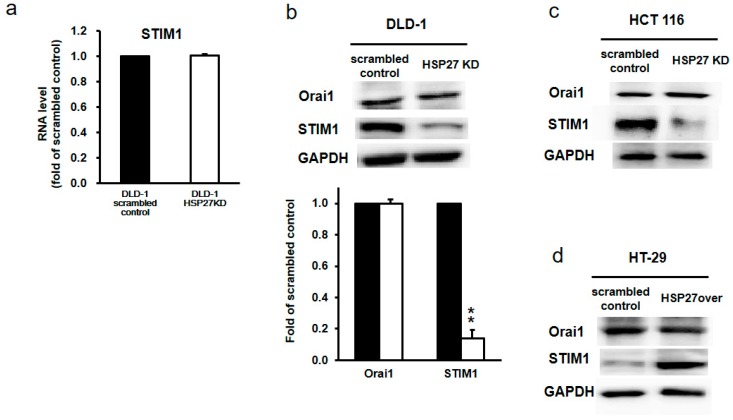
HSP27 increased the STIM1 protein levels in CRC cells. (**a**) QPCR was used to determine the STIM1 mRNA levels, and the levels were normalized to the GAPDH level. (**b**, **c** and **d**) Protein extracts prepared from the indicated cell lines were subjected to western blot analysis for STIM1 or Orai1. GAPDH was used as an internal control. Data are representative of three independent experiments. Values are the mean ± SD of three independent experiments. ** *p* < 0.01.

STIM1 and Orai1 are key proteins in mediating calcium channels. Therefore, we further assessed the levels of STIM1 and Orai1 in scrambled control and HSP27KD cells by qPCR and western blotting. Interestingly, the transcript levels of STIM1 and ORAi1 were similar in scrambled and HSP27KD cells (Figure 7a). However, the protein level of STIM1, but not Orai1, was decreased dramatically (Figure 7b,c). Overexpressed HSP27 in HT-29 cells resulted in an increase in STIM1 protein levels (Figure 7d). These results indicate that HSP27 level may influence the STIM1 protein levels. 

### 3.10. HSP27 Expression Maintained the Stability of STIM1 Proteins

**Figure 8:**
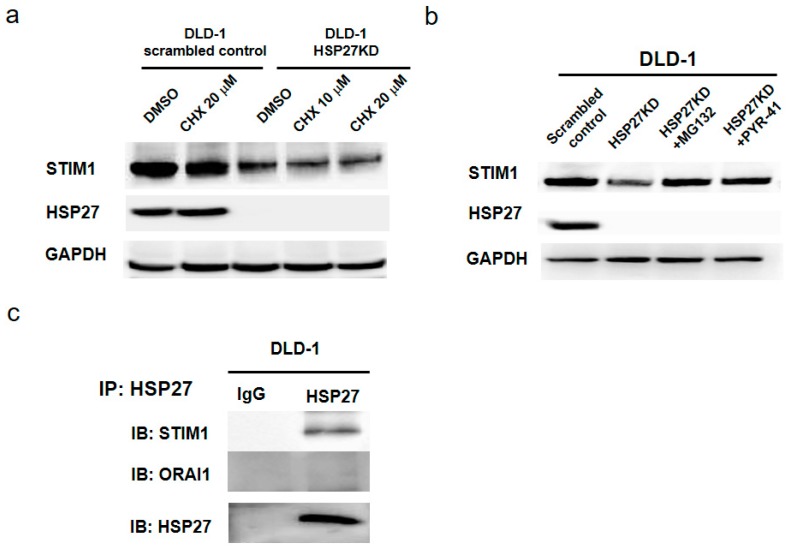
HSP27 prevented STIM1 degradation by directly binding to STIM1. (**a**,**b**) Scrambled control or HSP27KD cells were treated with cycloheximide (CHX), MG132 or PYR-41. Whole cell lysates were immunoblotted for STIM1 and HSP27. GAPDH was used as an internal control. (**c**) The possibility of direct protein-protein interactions was examined by immunoprecipitation (IP). All the experiments were independently performed at least 3 times.

To determine why silencing of HSP27 reduced STIM1 levels, we pretreated scrambled control and HSP27KD cells with cyclohexamine (CHX) and measured the protein level of STIM1. As shown in Figure 8a, the level of HSP27 was not affected in CHX-treated scrambled control cells. In HSP27KD cells, the level of STIM1 was lower than that in scrambled control cells. After pretreatment with CHX, the levels of STIM1 were reduced in a dose-dependent manner. These results indicate that low levels of STIM1 in HSP27KD cells may be due to a reduction in protein stability. To further confirm this hypothesis, we pretreated HSP27KD cells with a proteasome degradation inhibitor (MG132 or PYR-41) and found that pretreatment with MG132 or PYR-41 increased the STIM1 levels (Figure 8b). Together, these results indicate that the reduction of STIM1 in HSP27KD cells may be due to the proteasomal degradation pathway. 

### 3.11. STIM1 Was the Client Protein of HSP27

HSP27 is a chaperone that maintains protein stability and correct folding. To prove that HSP27 interacts with STIM1 to maintain the protein stability, we performed immunoprecipitation of DLD-1 cells. Cell extract from DLD-1 cells was incubated with HSP27 antibody overnight. Then, protein A beads were added to pull down the complex of the HSP27 antibody. As shown in Figure 8c, HSP27 interacted with STIM1 but not Orai1. These results demonstrated that HSP27 may help maintain the stability of STIM1. 

### 3.12. Silencing HSP27 Caused a Decrease in Puncta Formation of STIM1 under TG Induction

**Figure 9:**
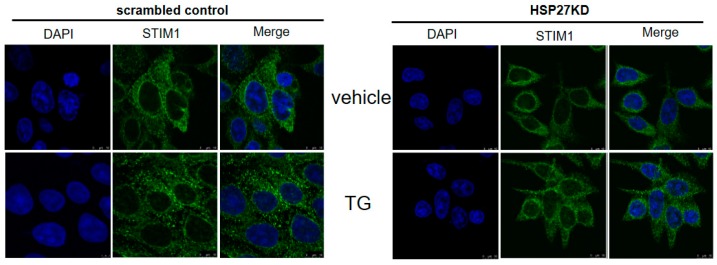
HSP27 promoted STIM1 oligomerization in DLD-1 cells. Scrambled control or HSP27KD DLD cells were seeded into coverslips and then treated with vehicle or TG for 15 min. Cells were fixed, and the distribution of STIM1 was detected by immunocytochemical staining. Confocal microscopy was applied to observe the distribution of STIM1 (green). Nuclei were identified by DAPI. All experiments were independently performed at least 3 times.

We further determined whether HSP27 is involved in the maintenance of STIM1 function in cells by assessing puncta formation. As shown in Figure 9, at baseline, the aggregation of STIM1 was decreased in HSP27KD cells. Moreover, after TG treatment, the oligomerization and aggregation of STIM1 were dramatically increased in scrambled control cells, and the STIM1 distribution showed little change in HSP27KD cells. These results suggest that HSP27 is necessary for the puncta formation of STIM1. 

### 3.13. HSP27 Was Associated with STIM1 Expression in CRC Specimens

**Figure 10:**
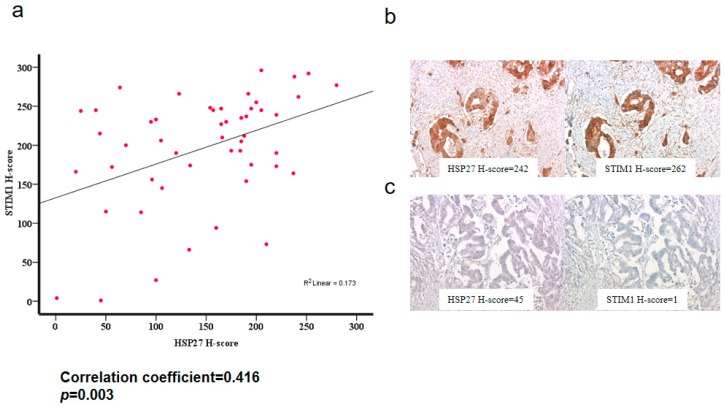
The association between HSP27 and STIM1 expression. (**a**) The scatterplot of H-scores of HSP27 and the corresponding STIM1 expression displayed a positive correlation (correlation coefficient = 0.416, *p* = 0.003). (**b**) A case of colon adenocarcinoma with a high HSP27 H-score displayed a high STIM1 H-score. (**c**) A case of colon adenocarcinoma with a low HSP27 H-score displayed a low STIM1 H-score.

To evaluate the association between HSP27 and STIM1 expressions, we performed HSP27 and STIM1 immunostaining on the other tissue microarray of colon adenocarcinoma. A positive correlation was observed between HSP27 and STIM1 expression levels (correlation coefficient = 0.416, *p* = 0.003) (Figure 10).

## 4. Discussion

**Figure 11:**
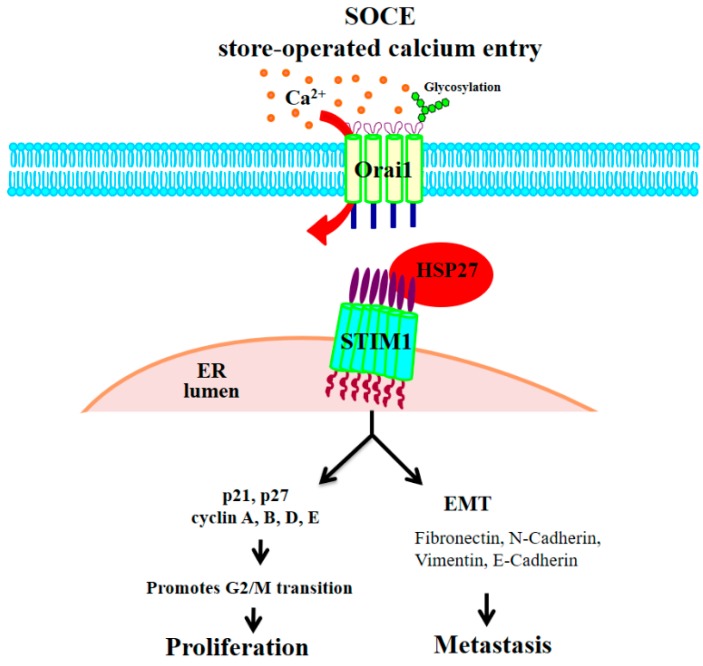
The proposed model for the role of HSP27 in CRC progression. HSP27 may interact with STIM1 to maintain the stability of STIM1. Silencing HSP27 caused a reduction of STIM1 to regulate the growth activity and metastasis ability on CRC.

CRC is one of the most common and deadly malignancies in the world, and diagnosis and treatment of this disease are a challenge. HSP27 acts as a chaperone to stabilize proteins in stressful conditions [44]. In cancer, high levels of HSP27 were associated with cell proliferation, anti-apoptosis effects, chemoresistance and poor disease outcome [45,46]. Recently, we demonstrated that HSP27 mediates progression and metastasis in liver cancer [35]. However, the role of HSP27 is still unclear. This study showed that in CRC, HSP27 not only affects proliferation, migration and invasion but also affects the cell cycle by promoting the G2-M phase transition and the EMT pathway and is associated with poor overall survival. Interestingly, HSP27 may play a role in the Ca^2+^ influx signaling pathway via the stabilization of the STIM1 protein (Figure 11). The levels of STIM1 and HSP27 were highly correlated in CRC specimens. These findings indicate HSP27 may be a new therapeutic target for CRC.

Ca^2+^ influx signals are believed to affect CRC progression, regulation of tumor migration and motility, cancer cell death and apoptosis p [47]. STIM1 and Orai1 are involved in the regulation of the mechanism of Ca^2+^ signaling, the SOCE. STIM1 acts as Ca^2+^ sensor on the endoplasmic reticulum (ER). The decreased calcium concentration in the ER is sensed, and STIM1 clusters forms “puncta” and relocated near the cell membrane, where it interacts with Orai1. Thus Orai1 Ca^2+^ channels on cell membrane activated with the internal calcium depletion [48,49]. STIM1 has many roles in tumorigenesis, as its silencing suppressed tumor migration or metastasis in breast, cervical, prostate, colorectal, brain, skin and liver cancers [32,33,34]. Targeting of either STIM1 or SOCE or both also influenced the sensitivity of cancer to different chemotherapeutic drugs. Silencing of STIM1 or SOCE promoted apoptosis induced by cisplatin in non-small cell lung cancer cells [50]. Targeting STIM1 or SOCE may sensitize prostate and human osteosarcoma cells to cisplatin treatment and hepatocarcinoma to 5-fluorouracil treatment by increasing autophagy, and overexpression of STIM1 increased chemoresistance in cisplatin-resistant cells [51,52]. However, no reports have examined the role of HSP27 in the Ca^2+^ influx complex. Protein level of ORAI1 showed no significant change with HSP27 KD. Silencing HSP27 reduced the protein level of STIM1 but no influence in mRNA level. We further checked with proteasome degradation inhibitors and our data indicates that HSP27 interacts with STIM1 to maintain its stability in CRC cells. Silencing HSP27 impaired the ability of STIM1 distribution and “puncta” formation. This is the novel finding in our study. This is the first report to show HSP27 may play the role in STIM1-mediated Ca^2+^ influx signals.

We demonstrated that targeting HSP27 selectively affects STIM1, which regulates and coordinates Ca^2+^ entry into the tumor cells, but not Orai1. HSP27 was also shown to be co-immunoprecipitated and stabilized STIM1 in this study, confirming the role of this gene as a chaperone. We restored the level of STIM1 in HSP27KD cells to determine whether the reduction of proliferation and migration could be reversed. However, we did not observe a reversal after overexpression of STIM1 in HSP27KD cells (data not show). These results indicate that maintenance of HSP27 level may be the essential factor for regulation of CRC. 

High levels of HSP27 in CRC specimens are associated with poor prognosis and have been classified as an independent prognostic factor [53]. However, it is unclear how HSP27 contributes to CRC progression. Our results demonstrated that HSP27 knockdown in CRC cells decreased cell growth through the arrest of the G2/M transition phase (Figure 3a,b). Then, we demonstrated that HSP27 silencing-induced G2/M phase arrest was due to changes in cell cycle-associated molecules (cyclin A2/B/D, p21 and p27) (Figure 3c,d). The cell cycle is a highly regulated process with checkpoint molecules that cause cell cycle arrest or activation [54]. In cancer, DNA repair after damage mainly depends on the G2 checkpoint, and many targets for the G2/M phase checkpoint are under development [55,56]. We showed that altered HSP27 levels may interfere with cell cycle regulation and then influence cancer cell survival. Therefore, specific targets or inhibitors of HSP27 could improve or complement current therapies by offering alternatives for CRC treatment. 

In summary, we demonstrated for the first time that HSP27 is a chaperone that stabilizes STIM1 of the SOCE, and its targeting resulted in arrest of G2/M transition, leading to decreased cancer proliferation, migration and invasion of colon cancer cells (Figure 11). These results indicate that HSP27 might be a promising therapeutic target for CRC.

## Figures and Tables

**Table 1:** Primer sequences used QPCR analysis with gene name.

Gene	Tow Way Direction	Sequence	TM °C
*STIM1*	forward reversed	5′-CTGGGATCTCAGAGGGATTTGA-3′ 5′-GCTGGCGGTCACTCATGTG-3′	60
*Orai1*	forward reversed	5′-GCTCATGATCAGCACCTGCAT-3′ 5′-GGGACTCCTTGACCGAGTTG-3′	60
*GAPDH*	forward reversed	5′-ATACTCCTGCTTGCTGATCC-3′ 5′-CCTGTACGCCAACACAGTGC-3′	60

**Table 2:** Association between clinicopathologic characteristics and HSP27 expression in colon adenocarcinoma.

Characteristics	HSP27 Expression *		*p* Value
	Low (*n* = 83)	High (*n* = 94)	
**Gender**			0.891
Male	45 (54%)	50 (53%)	
Female	38 (46%)	44 (47%)	
**Mean age (years) ± SD**	66.6 ± 11.4	66.7 ± 10.1	0.974
Age			0.831
<65 years	34 (62%)	40 (51%)	
≧65 years	49 (38%)	54 (49%)	
**Grading of carcinoma**			0.458
Well differentiated	7 (8%)	7 (8%)	
Moderately differentiated	53 (64%)	68 (72%)	
Poorly differentiated	23 (28%)	19 (20%)	
Invasive depth of tumor **			0.009
T1 + T2	8 (10%)	1 (1%)	
T3 + T4	74 (90%)	93 (99%)	
**Lymph node metastasis**			0.848
Negative	55 (66%)	61 (65%)	
Positive	28 (34%)	33 (35%)	
**Distant metastasis**			0.877
Negative	80 (96%)	91 (97%)	
Positive	3 (4%)	3 (3%)	

* Low expression: weakly positive HSP27 expression, high expression: moderately or strongly positive HSP27 expression. ** Missing data in 1 case.

## Abbreviations

CRC, colorectal cancer; HSP27, heat shock protein 27; STIM1, stromal interaction molecule 1; SOCE, store-operated calcium entry; ER, endoplasmic reticulum.
